# The President's Address

**Published:** 1901-08-03

**Authors:** 


### THE PRESIDENT'S ADDRESS.

The President's Address was delivered on Tuesday by George Bagot Ferguson, M.A., M.D., B.Ch.Oxon, who took for his subject Scientific Research: the Indispensable Basis of All Medical and Material Progress. In welcoming the members to Cheltenham, he said that the town, originally a royal manor of great antiquity, was so far as its present aspect was concerned comparatively modern, and owed its popularity to two causes—its water and its educational establishments. "You will notice," he said, "how we lie on a gentle slope of the Cotswolds with a natural drainage towards the Severn. You will see how sheltered we are on the east, and how open to the north and west; whilst from most directions the air blows on us, fresh and pure, either from the open wolds of the neighbouring hills, or from the valleys of Evesham and Gloucester. Our water supply, mainly from the hills, is pure and good. It contains lime, but not enough to produce calculous disorders, though sufficient to supply a needful constituent for growth and nutrition, so as to constitute one great factor of the high salubrity of Cheltenham, and of its reputation as a home for youth."

Referring to the Cheltenham waters, he thought their constitution was nearly the same as those of Carlsbad, with the exception that the last were hot and contained, in addition, more carbonate of soda and carbonic acid, a deficiency easily supplied by the addition of a little hot Vichy or Vals water, and thus they obtained a curative means of even greater efficacy in some cases.

Eighty years ago all the celebrities went to Cheltenham, but although they still had the waters with them the celebrities came no longer, but went to Carlsbad or Homburg, where their amusements at any rate were much better provided for. The use of the Cheltenham waters was, he said, chiefly for affections of the stomach and liver, and their efficacy in the diseases of Anglo-Indians had made the place one of their specially-favoured resorts. The principal wells had now passed into the keeping of the Corporation, the greatest care was taken to ensure the purity and uniformity of their yield, and he could assure all gastric and hepatic sufferers that if they would only drink the Montpellier saline or the No. 2 Pittville, warm, as they do the similar waters on the Continent, early in the morning in repeated small draughts, taking exercise between them, they would find them not one whit less efficacious than the very best of the springs of Germany. He further pointed out that the analyses of these waters showed them to be immeasurably superior to most of the German springs, to Homburg and Wiesbaden, to Kreuznach and Kissengen for instance, while in Nos. 1 and 3 Pittville we had an extremely salt water almost identical with that of Wiesbaden.

Dr. Ferguson then mentioned some of the medical worthies of the past connected with the town, after which he turned to the proper subject of his address, starting with the foundation of medicine and passing in review the many scientific discoveries which had brought medicine to the position it occupied to-day. Naturally in an address delivered in Gloucestershire the subject of vaccination occupied a prominent position. After speaking of Jenner, his great discovery, the honours done to him by other countries and the tardy and unwilling acceptance of his teaching by his own, Dr. Ferguson proceeded: "Later came detraction, obloquy and neglect, followed at last by 443 smallpox deaths in 1896 in his own county in the neighbouring city of Gloucester; and finally the perilous experiment of leaving the decision as to whether vaccination should be done or not to the discretion or indiscretion of each head of a family. . . . Truly you will mourn with me over those 443 preventable deaths in Gloucester, one of them of a vaccinated and 297 of them of unvaccinated children under 10 years of age, when you reflect that during the same year and the next one there were in well-vaccinated and revaccinated Germany only 15 deaths from smallpox throughout a population of 53 millions." Turning finally to the question of education he compared the scientific enthusiasm everywhere manifested in other countries with the anti-scientific spirit which characterised most of the wealthier and more cultivated classes in this country, and urged that the question of University Education was a matter of life and death for this country.

